# Maritime route and vessel tracklet dataset for vessel-to-route association

**DOI:** 10.1016/j.dib.2022.108513

**Published:** 2022-08-04

**Authors:** Clément Iphar, Anne-Laure Jousselme, Giuliana Pallotta

**Affiliations:** a NATO STO Centre for Maritime Research and Experimentation, La Spezia, Italy; b Lawrence Livermore National Laboratory, Livermore, CA, United States

**Keywords:** Automatic identification system, Vessel-to-route association, Track-to-route association, Maritime routes, Route prototypes

## Abstract

With an ever-increasing number of vessels at sea, the modelling, analysis and visualisation of maritime traffic are of paramount importance to support the monitoring tasks of maritime stakeholders. Sensors have been developed in this respect to track vessels and capture the maritime traffic at the global scale. The Automatic Identification System (AIS) is transmitting maritime positional and nominative information at highest frequency rate, making it a valuable source for maritime traffic modelling. From an original AIS dataset covering the area of Brest, France, we extracted a set of 17 maritime routes, connecting ports in this area. Two different representations for the routes are provided: (1) clusters of AIS contacts, and (2) route prototypes, representing the nominal trajectory of the vessels following the route. Additionally, a set of tracklets (built by five consecutive AIS contacts from the same vessel trajectory) has been extracted from the set of routes and the original dataset, and labelled either with the route name to which they belong or as off-route tracklets. This dataset provides thus some ground truth on the routes followed by vessels and is aimed at testing and validating vessel-to-route or track-to-route association algorithms.

## Specifications Table

**Table:** 

Subject	Maritime Mobility and Transport
Specific subject area	Generation of maritime routes and of their prototypes, association of vessel tracklets to maritime routes
Type of data	Data files: ship positions; maritime routes; tracklets; route information
How the data were acquired	Original data: AIS data from [1] Maritime routes (clusters): TREAD (Traffic Route Extraction and Anomaly Detection) [2] software, in MATLAB Navigation tracklets: tracklet extraction software, in R Route prototypes: prototype generator software, in MATLAB Route nomenclature: generated from computation output Ports of interest: partially retrieved from [3]
Data format	Raw: navigation tracklets Filtered: ship positions and kinematic details; maritime routes
Description of data collection	From [1] dataset, the TREAD software has been run on 6 months of data, producing routes as clusters of contacts. Only relevant routes (i.e., with enough data contacts) are kept. 800 ground truthed 5-points maritime tracklets are extracted from this set of routes, and removed from the route cluster they belong to. Route prototypes are computed as synthetic trajectories from the remaining points in the cluster for each route.
Data source location	Celtic Sea, North Atlantic Ocean, English Channel, Bay of Biscay (mainly France, marginally United Kingdom)
Data accessibility	Zenodo deposit [4], named “Maritime routes and vessel tracklet dataset for vessel-to-route association”, Available at https://doi.org/10.5281/zenodo.6402160 Usage rights: Creative Commons Attribution-NonCommercial-ShareAlike 4.0 International (CC BY-NC-SA 4.0) Repository name: Zenodo Data identification number: 6402160 Direct URL to data: https://doi.org/10.5281/zenodo.6402160

## Value of the Data

• The maritime route and vessel tracklet dataset provides at once (1) a set of maritime routes computed from a reference benchmark AIS dataset, together with (2) a set of labelled maritime tracklets. Such a combination provides ground truth information about the route followed by the vessels. The objects of maritime routes and their two representations (clusters and prototypes) are unique and allow testing algorithms for associating vessels to routes;
• Research on maritime route extraction, maritime situational awareness, track-to-route association, vessel destination prediction can benefit from these data. Besides research purposes, operational purposes include maritime surveillance, security and safety of navigation, anomaly detection and any application requiring estimating the origin or the destination of a vessel;
• The general data extraction method described in this paper enables the extension of the heterogeneous and integrated dataset [1] with maritime routes and ground truthed tracklets datasets. While the method for generating maritime routes can be chosen from any available in the literature, the specific method for ground truthed tracklets generation is original. Moreover, it is *independent* of the route extraction method and can thus be applied to any previously extracted maritime route dataset. Furthermore, it can be used to generate data for new areas or different time periods from the AIS dataset from [1].;
• The maritime route and vessel tracklets dataset provides a support for understanding maritime activities and for the development of models that require the vessel (and its track) to be associated, either fully or partially, to maritime routes. Further local insights can be found about the maritime traffic, estimating the possible routes a vessel could follow and the potential destination ports, thus enabling route prediction and, potentially, anomaly detection;
• This dataset provides the maritime routes in both point cluster and route prototype representations, enabling the development and testing of various algorithms on the same set of maritime routes.

## 1 Data Description

The maritime route and vessel tracklets dataset is composed of five data files: (1) the maritime routes represented as point clusters, (2) the maritime routes under prototype forms, (3) the vessel tracklets and corresponding labels, (4) the route nomenclature and (5) the names of ports of interest. Table 1 presents some characteristics of the five data files. The total size of data is 819 Kb.

**Table 1:** List of the data files of the maritime route and vessel tracklets dataset.

File	File name	Size	# rows	Type	Sep.	SRID	Licence
Point cluster maritime routes	all_points.csv	565 Kb	11381	CSV	|	4326	CC-BY-NC-SA-4.0
Route prototypes	prototypes.csv	3 Kb	121	CSV	|	4326	CC-BY-NC-SA-4.0
Tracklets and labels	tracklets.csv	250 Kb	800	CSV	|	4326	CC-BY-NC-SA-4.0
Routes nomenclature	nomen.csv	0.5 Kb	17	CSV	|	4326	CC-BY-NC-SA-4.0
Ports of interest	poi.csv	0.3 Kb	7	CSV	|	4326	CC-BY-NC-SA-4.0

In the remainder of this section, each file will be presented in Tables 2, 3, 4, 5 and 6. Each data feature, corresponding to the columns in the CSV files, are shown in each row of Tables 2, 3, 4, 5 and 6, specifying their nature, the values they can take and a short description. The files of the routes under cluster and prototype forms together with the labeled tracklets are the core of this dataset, with the files on route nomenclature and ports of interest coming as complementary information to enable a larger use of this dataset.

### 1.1 Route clusters

The point cluster maritime routes data file contains points that belong to the cluster of the 17 maritime routes. Each of the 17 routes is defined by a cloud of points, each point being an AIS contact of a vessel that followed that route. Each row of the file corresponds to one contact in the cluster. The feature **idpoint** is the primary key of the table and once the points have been sorted by ascending value of their primary key, the rows are ordered by route first (from R_01 to R_17) and then arbitrarily by ascending longitude value. The total number of points (rows) is 11,381, unevenly distributed across routes as shown in Fig. 3. Table 2 details the characteristics of the fields (columns) of this file.

**Table 2:** Description of the data features of the “Point clusters” data file

Feature	Type	Values	Short description
idpoint	Integer	[1,11378]	Point identifier, primary key
longitude	Real, 6 decimals	]-180, 180] U {181}	Longitude of the point (WGS84)
latitude	Real, 6 decimals	]-90, 90] U {181}	Latitude of the point (WGS84)
sog	Real, 1 decimal	[0, 102.4]	Speed over ground at that point
cog	Real, 1 decimal	[0, 359.9]	Course over ground at that point
ts	Integer	[1443657600, 1459468800]	Message timestamp at that point
route	Text	{R_1, …, R_17}	Route to which the point belongs

### 1.2 Route prototypes

The route prototype data file contains all points that belong to the computed prototypes of the maritime routes. This table represents the same element as the cluster of points (the maritime routes) under a different form. Indeed, under the form of synthetic track, the 17 routes are defined as an ordered set of spatial points. The primary key of this table is a composite key of two features: the route column and the number column. The number column represents the ordering of the point in the track, which is fundamental, as a track is an ordered succession of points. The total number of points is 121, each prototype trajectory being defined by a number of points ranging from 3 to 11.

**Fig. 1:**
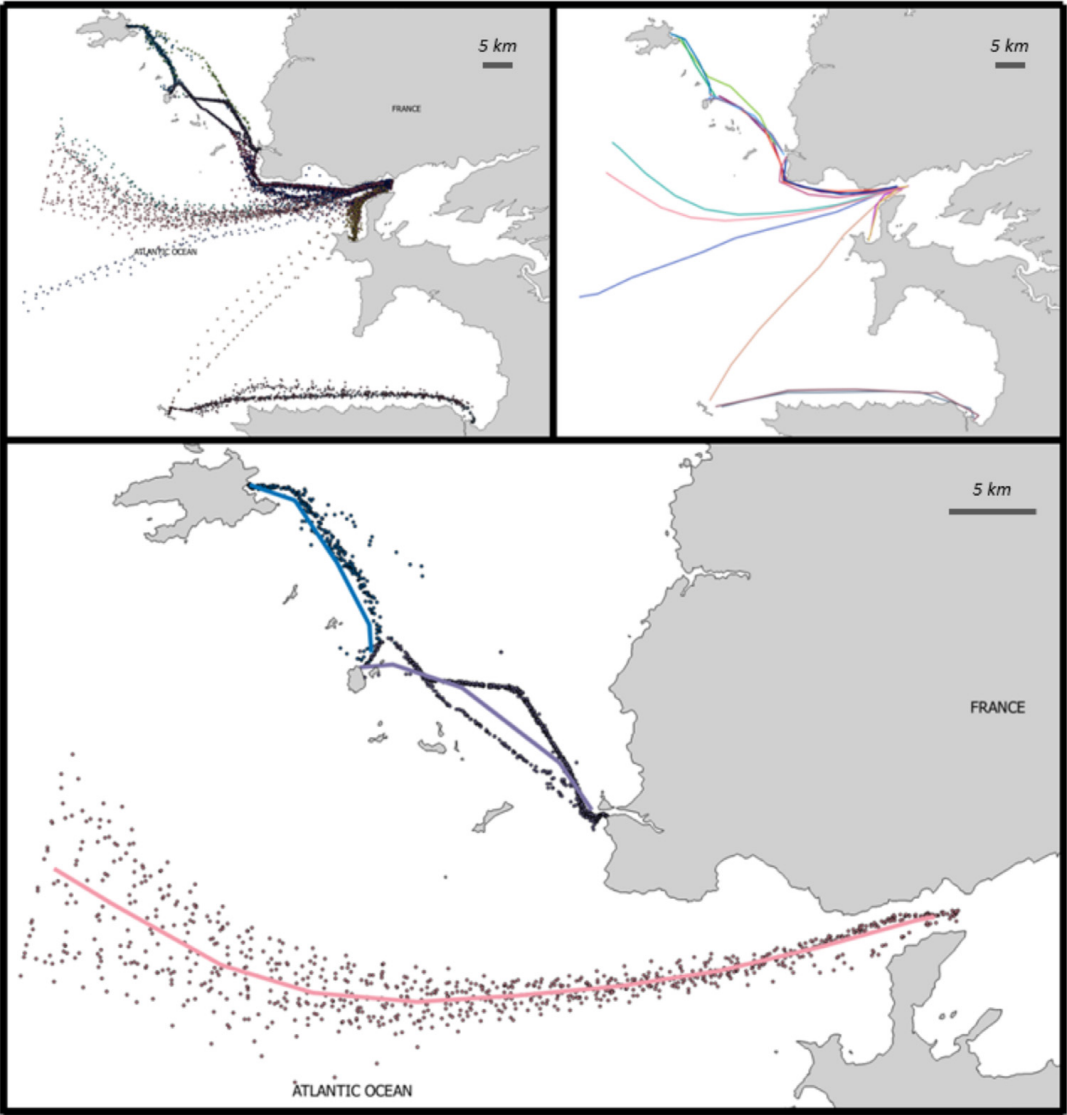
Computed maritime routes with different representations. Each route has a different colour. The route prototypes are directional, this is not shown in this graphical representation but can be deduced from the route nomenclature. Top-left: representation of the 17 routes under the form of a cloud of points. Top-right: representation of the 17 routes under the form of prototype trajectories. Bottom: Zoom on a selection of three routes and superposition of the two representations.

Fig. 1 displays the route prototypes, the route point clusters and illustrates the different representations of the same object. Table 3 details the characteristics of the fields of this file.

**Table 3:** Description of the data features of the “route prototype” data file

Feature	Type	Values	Short description
Route	Text	{R_01, …, R_17}	Route to which the track belongs
pointnumber	Integer	[1,11]	Rank of the point in the track
longitude	Real, 6 decimals	]-180, 180] U {181}	Longitude of the point (WGS84)
latitude	Real, 6 decimals	]-90, 90] U {181}	Latitude of the point (WGS84)

### 1.3 Labelled tracklets

The labelled tracklets data file gathers all maritime tracklets extracted from the set of maritime routes and the original AIS heterogeneous integrated dataset [1]. The tracklets consist of five consecutive AIS contacts (or points) with their respective kinematic and identity feature values (speed and course over ground, MMSI), complemented by a label to be described below. The 800 tracklets are divided into two categories: 400 “on-route” tracklets (with **idtracklet** in [1, 400]) and 400 “off-route” tracklets (with **idtracklet** in [401, 800]). For the on-route tracklets, the value of the route feature is set to the name of the route the tracklet has been extracted from (route labels go from R_01 up to R_17, *cf.* Table 5), and for the off-route tracklets, the value of the route feature is set to the “no route” labelled as R_0. Table 4 details the characteristics of the fields of this file.

**Table 4:** Description of the data features of the “Maritime tracklets” data file

Feature	Type	Values	Short description
Idtracklet	Integer	[1,800]	Tracklet identifier, primary key
idX	Integer	[1, 18,648,556]	Unique identifier of the point n°X
mmsiX	Integer	[10^8,10^9-1]	MMSI value for point n°X
speedX	Real, 1 decimal	[0, 102.4]	Speed over ground of point n°X
courseX	Real, 1 decimal	[0, 359.9]	Course over ground of point n°X
headingX	Integer	[0, 359] U {511}	Value of heading for point n°X
lonX	Real, 6 decimals	]-180, 180] U {181}	Longitude (WGS84) of point n°X
latX	Real, 6 decimals	]-90, 90] U {91}	Latitude (WGS84) of point n°X
tsX	Integer	[1443657600, 1459468800]	Message timestamp of point n°X
Route	Text	{R_0, R_01, …, R_17}	Route from which the tracklet is extracted

**Fig. 2:**
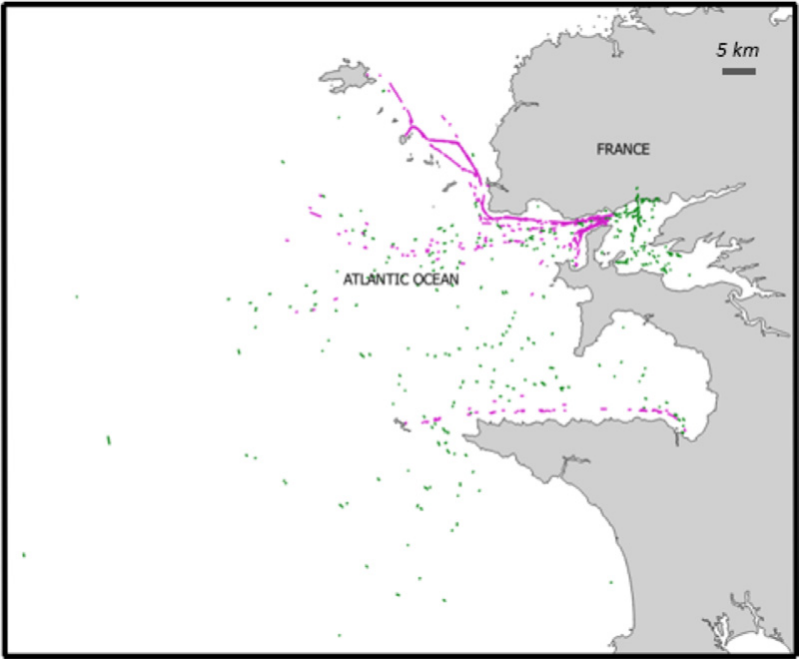
Maritime tracklets extracted from the maritime routes. Purple: “on-route” tracklets, Green: “off-route” tracklets

**Fig. 3:**
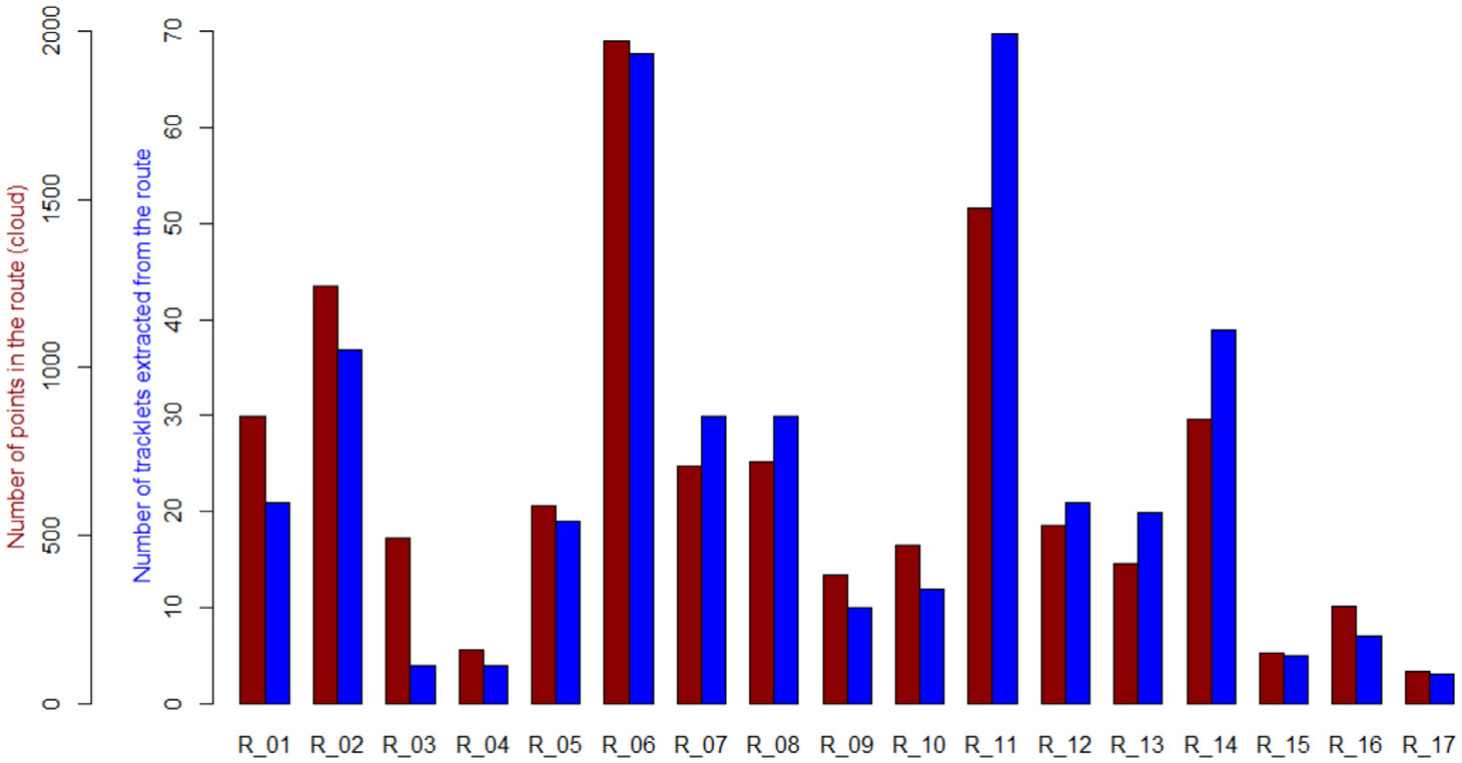
By route, number of points in the point cluster (red) and number of tracklets extracted (blue).

The value X shown in rows 2 to 9 of Table 4 takes values in {1, 2, 3, 4, 5}. Therefore, the total number of features in the file is 42: **idtracklet**, 5 times 8 points and the route label. The partition of the 400 on-route tracklets on the set of 17 routes is shown in Fig. 2. The number of tracklets extracted by route is displayed in Fig. 3.

### 1.4 Route nomenclature

The route nomenclature file provides basic information about each route, with the usual name of both the port of origin and the port of destination. This file has 17 rows, and the primary key is the route feature, consisting of the code name of the route. It is referenced as foreign key for the three route features of the route prototype, cluster of points and maritime tracklets tables. Both **originport** and **destinationport** features reference the **portname** column of the port of interest table as a foreign key. The length of the route is defined as the sum of the lengths of its route prototype segments. The great arc length of each segment is computed using the Haversine formula, taking as local radius of the Earth the radius at latitude 48.5°. When the port of Brest is involved, the track stops at the entrance of the roadstead. The offset between the entrance of the roadstead and the entrance of the port is ca. 5.6 km. Table 5 details the characteristics of the fields of this file.

**Table 5:** Description of the data features of the “Route Nomenclature” data file

Feature	Type	Values	Short description
Route	Text	{R_01, …, R_17}	Route of interest
Originport	Text	{Set of Ports}	Usual name of the port of origin
destinationport	Text	{Set of Ports}	Usual name of the port of destination
Length	Integer	N	Length in meters of the synthetic track, computed by addition of loxodromic distances

### 1.5 Ports of interest

**Fig. 4:**
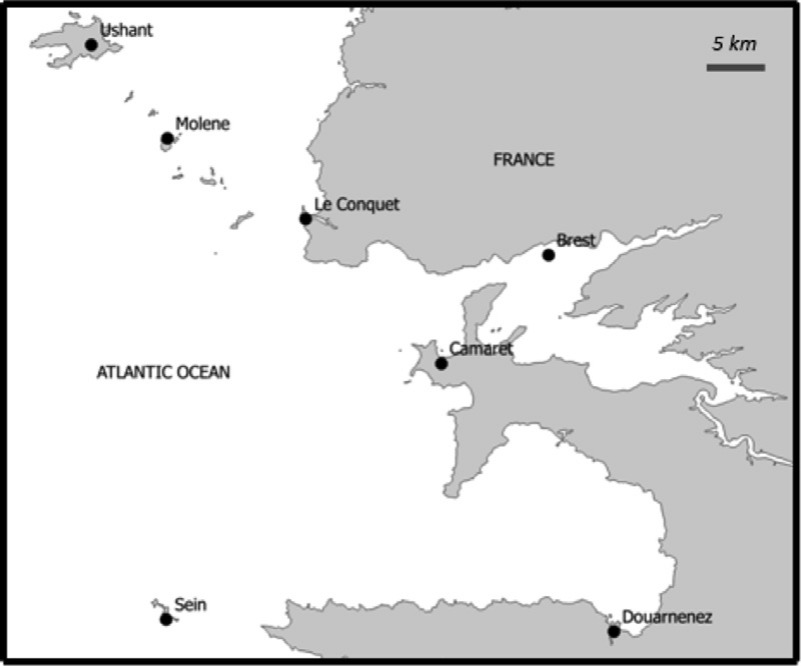
Location of the ports of interest

The port of interest data file provides information about the ports of interest, which are all ports for which at least one of the 17 routes is either an origin or a destination. The primary key of this table is the feature **portname**, which is the usual name of the port, and additional features are the location of the port, its official name and LOCODE [3]. Table 6 details the characteristics of the fields of this file, and the location of all 7 ports of interest is shown in Fig. 4.

**Table 6:** Description of the data features of the “Ports of interest” data file

Feature	Type	Values	Short description
portname	Text	{Set of Ports}	Common name of the port of interest
offname	Integer	[1,11]	Official name of the city where the port of interest is located
longitude	Real, 6 decimals	]-180, 180]	Longitude of the port (WGS84)
latitude	Real, 6 decimals	]-90, 90]	Latitude of the port (WGS84)
locode	text	5-characters codes	Official UN/LOCODE name of the port, if existent

## 2 Experimental Design, Materials and Methods

**Fig. 5:**
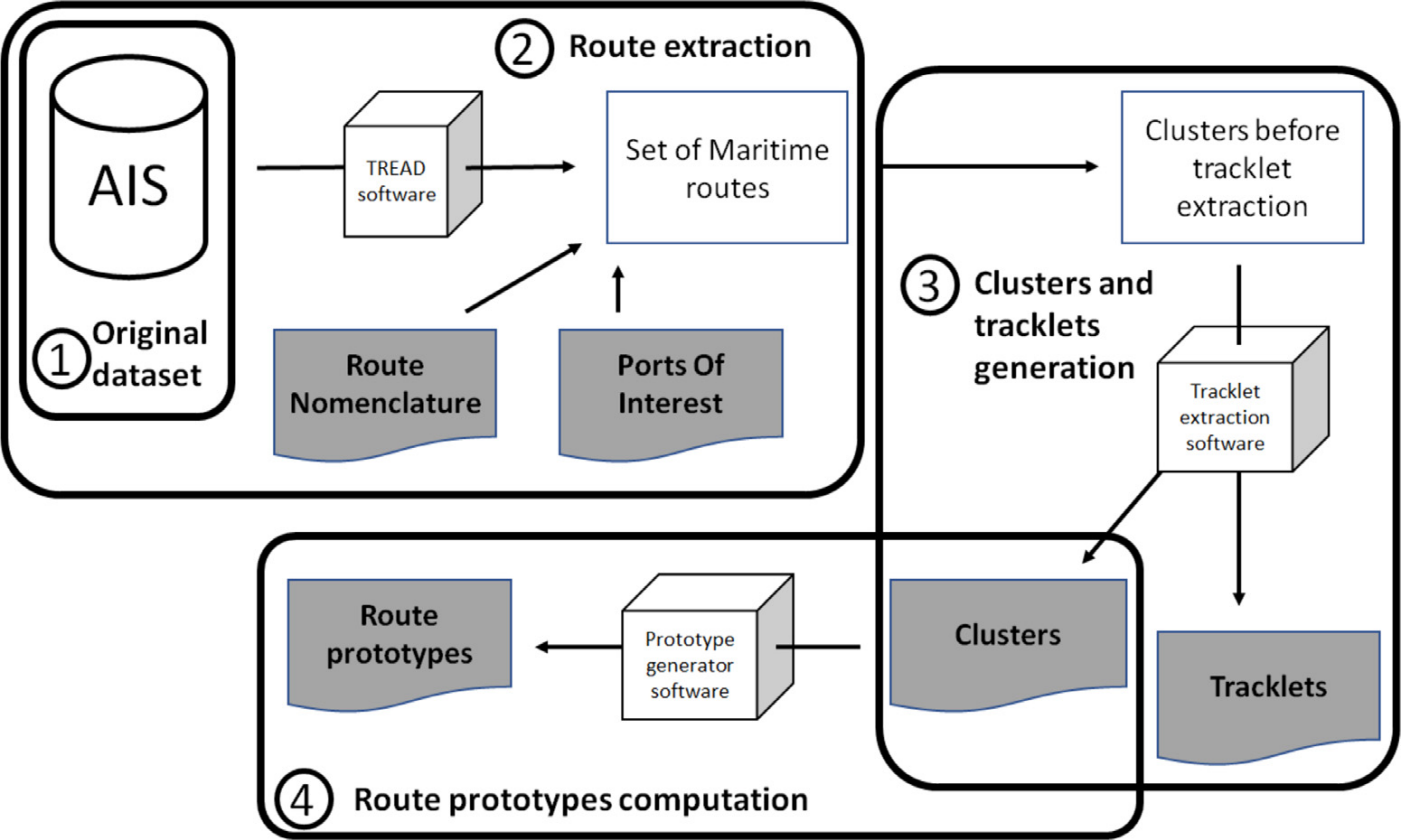
Workflow for dataset generation

Fig. 5 presents a synoptic schematic representation of the methods used for the generation of the maritime route and vessel tracklets dataset. The different parts will be further detailed in this section.

### 2.1 Original dataset

**Fig. 6:**
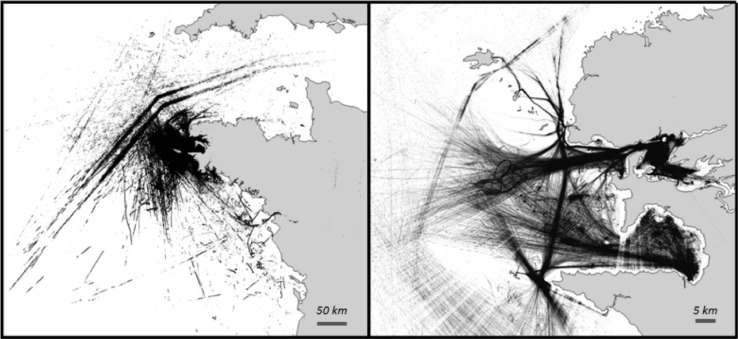
The dynamic AIS from the heterogeneous integrated dataset described in [1]. Left: general view. Right: close-up on the Brest area

The original AIS dataset discussed in this paper is excerpt from the heterogeneous integrated dataset published in [1], under the licence CC-BY-NC-SA-4.0. This heterogeneous integrated dataset features AIS dynamic information, AIS static information, cartographic information, excerpts from fleet registers, fishing areas, or navigation-related information. The dynamic AIS data consists of 18,648,556 messages, gathered in a unique flat file (CSV) dynamic messages defined by the International Telecommunication Union as messages number 1, 2, 3, 18 and 19. The AIS dataset covers the temporal period from October 1^st^ 2015 to March 31^st^ 2016, with whereabouts given in the WGS84 coordinate system. The messages were received by a single antenna and feature the MMSI (Maritime Mobile Service Identity, an international unique ship identifier), the coordinates, the speed over ground in knots, the true heading and the course over ground in degrees relative to the True North, the rate of turn in degree per minute and the navigational status (current motion status of the vessel). Since AIS messages do not embed the timestamp of emission, the timestamp of reception is added upon parsing with an UNIX Epoch integer. Fig. 6 shows the positional data of the original AIS dataset, each dot representing one data point. The antenna is located near Brest, France, and the data points are scattered over the reception area of the antenna, in the Brest roadstead, off the coast of Brittany, North towards Cornwall and South in the Bay of Biscay.

The maritime route and vessel tracklet dataset described in this paper has been built from an excerpt of the AIS heterogeneous integrated dataset described above, with the same timeframe but with the following spatial bounds: latitude between 47 and 50 degrees North, longitude between 7 and 1 degrees West.

### 2.2 Routes extraction

The methodology for generating the ground truthed tracklets datatset is independent from the maritime route extraction method. The TREAD (Traffic Route Extraction and Anomaly Detection) algorithm [2] to be used in this work and detailed in this section, is a precursor on the topic and inspired several works. It has been improved in [5] to build a maritime traffic network with synthetic representation reducing the storage space. The underlying *trajectory-based* approach used in TREAD is a step forward compared to another related papers using a *point-based* approach where AIS vessel messages are treated as disjoint points on a grid and are clustered together to reconstruct the vessel routes, as described in [6]. Another innovative aspect of TREAD approach, compared to other methods available, is the incremental application of DBSCAN (Density-Based Spatial Clustering of Applications with Noise) Algorithm in the extraction of routes, ports and entry/exit gates. As such, the methodology can build on top of the objects already been derived, by re-starting the clustering from the last derived set of routes and performing an incremental update of the learned significant objects. Starting from these concepts, [7], the authors propose a modified version of the DBSCAN) algorithm suited to areas with high density of vessels. In [8], the authors build a network for the maritime traffic defining semantic objects as trajectories and clustering waypoints and stopping points, while the method proposed in [9] uses itself genetic algorithms for clustering data points, and creates a directed graph to represent maritime routes. However, even in the most recent papers on route extraction from AIS data (e.g., [10]) the main underlying assumptions remain close to those initially presented in TREAD seminal paper [2].

In this section, we describe the method used for the generation of maritime routes from the original AIS heterogeneous integrated dataset. It has been conceived as an iterative process involving the TREAD software although it could be replaced by any other method, as the method for generating the tracklet is independent of the maritime route extraction method.

TREAD methodology is based on the incremental DBSCAN algorithm to process raw AIS data (both terrestrial and satellite data) to extract ‘patterns of life’ shaped as maritime routes, ports and entry and exit points within the selected bounding box.

In order to detect ports, stationary events are identified by speed gating based on the positional displacements of the vessel in two consecutive points. In this way the starting and ending points for each maritime route are derived and updated as soon as new data point becomes available (i.e., incremental clustering). Once the waypoints are learned, the route clusters can be derived by clustering the vessel flows, which connect two waypoints. The route point clusters are built by checking for all the vessel positions which passed from the starting and ending point in the selected time window. In the learning phase noise points as classified by the DBSCAN algorithm are discarded as they are considered outliers. Similarly, points showing inconsistencies (*e.g*., inexistent or inconsistent MMSI, unphysical position, etc.) are also discarded to provide a clean picture of the traffic in the area. All these discarded points are stored in a separate list, in case of need in a second time (*i.e*., when performing route prediction or anomaly detection).

Route clusters keep information about the transiting vessels, but also of the static and kinematic features of the vessels associated to them. There is no assumption about the number of routes to be automatically learned by TREAD.

A set of possible routes of interest has been first extracted after an initial run of the software on one month of data. Then, those routes were divided into categories, from the smallest to the largest, establishing a processing order for the route extraction.

**Fig. 7:**
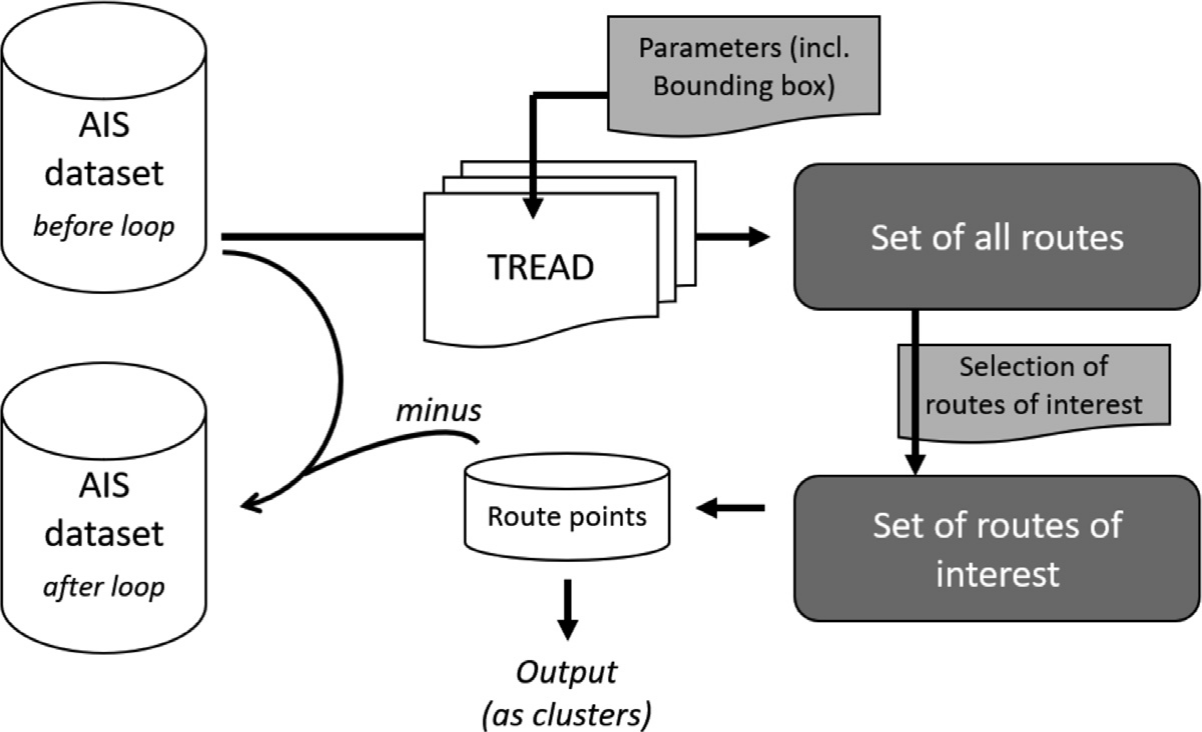
One loop of extraction of routes of interest, with modification of AIS dataset

Fig. 7 shows one iteration of the process, with the output of route clusters and the updated AIS dataset. The dataset is initialised by the original heterogeneous integrated dataset presented in Section 2.1 and continuously updated at each loop. The computational parameters for TREAD are modified at each loop: mainly the bounding box that allows focusing on specific areas where routes have previously been detected. This semi-manual refinement iterative process allows an improved quality of the routes (enough points, coherent spatial distribution), tuned to some variable granularity. At the last step of the loop, all data points that do belong to any of the selected routes from the dataset are removed, so that those points cannot be used to define other routes in subsequent loops.

Table 7 presents all the parameters (*i.e*., the bounding boxes) used at the different iterations, together with the number and names of routes extracted. All loops were run with the same timespan: all 6 months of data.

**Table 7:** List of the extracted routes by iteration

Iteration	lonmin	lonmax	latmin	latmax	nbroutes	Routes
1	-4.98	-4.76	48.34	48.42	2	[R_01,R_02]
2	-5.14	-4.93	48.38	48.50	2	[R_03,R_04]
3	-4.83	-4.54	48.29	48.37	2	[R_05,R_06]
4	-4.62	-4.54	48.27	48.37	2	[R_07,R_08]
5	-5.14	-4.76	48.34	48.50	1	[R_09]
6	-4.98	-4.54	48.29	48.42	2	[R_10,R_11]
7	-4.75	-4.28	48.08	48.34	0	
8	-4.89	-4.28	48.02	48.15	2	[R_12,R_13]
9	-5.14	-4.54	48.02	48.50	4	[R_14,R_15,R_16,R_17]

The 17 routes retained were named R_01 to R_17, with an arbitrarily numbering. Route nomenclature does not reflect any meaningful ordering for our purposes.

The route nomenclature table is manually generated, with only the feature “length” left filled at the route prototype computation stage. The Ports of Interest table is also manually generated and filled with relevant information about each port corresponding to either the origin or the destination for at least one route. The source for UN/LOCODE feature is [3].

### 2.3 Clusters and tracklets generation

The clusters and tracklets generation process takes as input the route points as given by the TREAD software and outputs both a ground truth table and the final point clusters for all routes.

The ground truth table consists of 400 on-route and 400 off-route tracklets, as defined in Section 1.1. On-route and off-route tracklets follow different paths for their generation, although they are eventually gathered into a single table.

As for the in-route tracklets, a uniform random draw without replacement is performed amongst the set of route points. The validity of the drawn point as a candidate for initiating a tracklet is then assessed according to the algorithmic procedure in Algorithm 1, following three steps. First, a point is valid if it is followed and preceded by two pairs of points, sent by the same vessel, so that we can extract a complete tracklet of 5 consecutive points. Second, a point is valid if the time difference between the first and the last message is below some threshold. Since a tracklet represents a subpart of a full track, it is important that its temporal span remains minimal. In this respect, a threshold value of $\tau_{on}$ has been set to 240 seconds. Third, the last verification consists in checking that none of the four other points belong to another tracklet. Note that the candidate cannot, since it has been drawn without replacement. If all three conditions are fulfilled, then the candidate point is deemed as valid, the tracklet is built adding the two preceding and following points, the name of the route to which the tracklet belongs is retrieved, as well as all relevant information to be inserted in the tracklet file (see Section 1.3).

**Algorithm 1:** Generation of on-route maritime tracklets.

As for the on-route tracklets, we follow an equivalent procedure for the off-route tracklets. A uniform random draw without replacement is performed amongst the set of all data points (from the original heterogeneous integrated dataset), restrained to spatial bounds (latitude between 47 and 50 degrees North, longitude between 7 and 1 degrees West) so that we avoid tracklets being drawn in anomalously far away points. In a similar way as for on-route tracklets, the validity of the candidate point is then assessed according to the algorithmic procedure shown in Algorithm 2. The validity of the candidate follows four steps. The first and second steps are the same as for the on-route tracklets. However, the threshold used in the second step could be different to allow more flexibility on long-range reception. The third step is also similar, but all five points are tested for their membership to any former tracklet (including the on-route tracklets). The fourth step consists in verifying if each data point that has the same MMSI as the candidate, within a temporal distance of one hour of the candidate. If any of those points is part of a route data cloud, then the tracklet is considered not being off-route, and is discarded as a candidate. If all four conditions are fulfilled, then the candidate is deemed as valid, and relevant information is retrieved to be inserted in the tracklet table, with the route field being set at $R_{0}$.

**Algorithm 2:** Generation of off-route maritime tracklets.

The computation of the on-route tracklets is performed before the computation of the off-route tracklets. For both on-route and off-route tracklets, when the number of valid candidates reaches 400, the computation is stopped. The tracklets are numbered, the values of the features of the 5 data points are retrieved and the tracklet is labelled either with the name of the route from which the point was drawn in the on-route case or with the $R_{0}$ value otherwise.

In addition, the five points building each valid tracklet of the on-route cases, are removed from the cluster of route points, if applicable. In each tracklet, point 3 is systematically removed from the cluster of route points, by construction (as it was drawn from this list). Out of the 400 tracklets generated, 412 points were removed, and the total number of lines in the cluster points table stands at 11,381.

### 2.4 Route prototype computation

This part of the process takes as input the set of points and outputs a route prototype under the form of a synthetic trajectory, and it is performed for each route individually. The piece of software to create the route prototype is iterative, following the temporal order of the points of the trajectory. The computation of the n points $P_{i},\;\forall i\in \left[1,n\right]$ of the set of points into a route prototype was resorting to finding, in an iterative way, the nearest points within a radius R to an initial synthetic point $\Gamma_{k}$ of latitude $\varphi_{k}$ and longitude $\lambda_{k}$. For the point $P_{i}$ to be picked, the distance $\Gamma_{k}P_{i}$ had to be shorter than R. The average in heading H and in speed S of all the picked points were computed to generate a new synthetic point $\Gamma_{k+1}$, located at the barycentre of all picked points.

Since we consider a trajectory as a forward motion, rather than considering a circle of radius R as the area of interest, we consider a semi-circle, centered on the local value of the heading, thus enabling to select only points that are ahead of the initial point, in the sense of motion.

As it might happen that the set of point is so scattered that no point is detected within the semi-circle of radius R, the original radius is incrementally multiplied by ascending naturals (2, 3, 4, …) until the semi-circle is not empty and a new step of the iteration becomes possible.

**Fig. 8:**
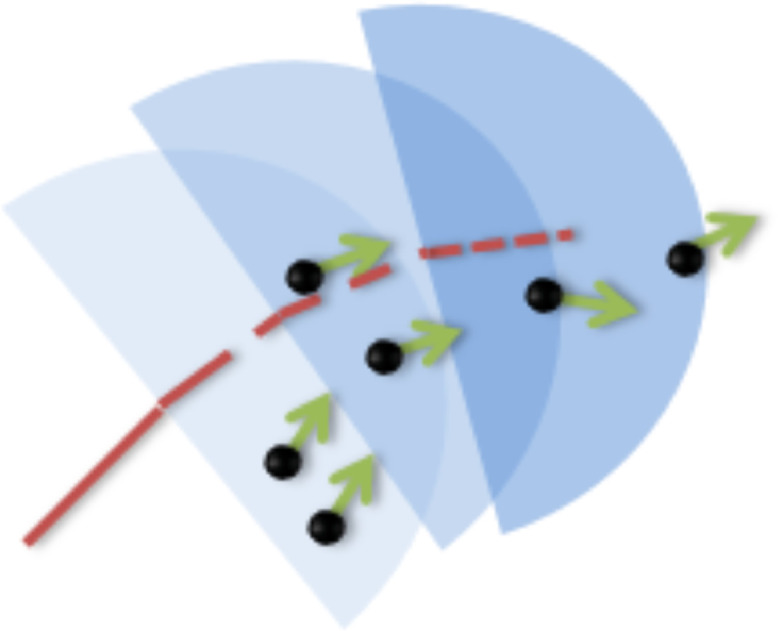
Iterations with semi-circle forward motion technique of [11]

The general principle of the iteration using semi-circle forward motion is shown in Fig. 8. The condition of belonging to the semi-plane of interest for the point $P_{i}$ is for the scalar product of $\Gamma_{k}P_{i}$ and $H_{k}$ to be positive.

The process is initialised at the coordinates of the port of origin, where a full circle is used rather than a semi-circle as no heading value is available. The initialisation point is not included in the route prototype. The process is ended once the semi-circle reaches the coordinates of the port of destination, and no synthetic point is computed in this case.

At each loop, the synthetic points that constitute the route prototype are computed as:

$$\Gamma_{k+1}\left[\varphi_{k+1},\lambda_{k+1}\right]=\bar{P}\left[\varphi_{I},\lambda_{I}\right],\;\text{where}\;\;I=\left\{i\right\},I\in \left[1,n\right]\;|\;\Gamma_{k}P_{i}\;<\;R,\;\Gamma_{k}P_{i}*H_{k}\;>\;0\;$$

$$\left[H_{k+1},S_{k+1}\right]=\left[{\bar{H}}_{I},{\bar{S}}_{I}\right],\;\text{where}\;I=\left\{i\right\},I\in \left[1,n\right]\;|\;\Gamma_{k}P_{i}\;<\;R,\;\Gamma_{k}P_{i}*H_{k}\;>\;0$$

## CRediT Author Statement

**Clément Iphar:** Data curation, Conceptualization, Methodology, Software, Writing – original draft preparation, Writing – review & editing; **Anne-Laure Jousselme:** Conceptualization, Methodology, Supervision, Writing – review & Editing; **Giuliana Pallotta:** Conceptualization, Software, Writing – review & editing.

## Declaration of Competing Interest

The authors declare that they have no known competing financial interests or personal.

## Data Availability

Maritime routes and vessel tracklet dataset for vessel-to-route association (Original data) (Zenodo).
